# Polycyclic aromatic hydrocarbon (PAH) source identification and a maternal transfer case study in threatened killer whales (*Orcinus orca*) of British Columbia, Canada

**DOI:** 10.1038/s41598-023-45306-w

**Published:** 2023-12-19

**Authors:** Kiah Lee, Stephen Raverty, Paul Cottrell, Zeinab Zoveidadianpour, Brendan Cottrell, Dana Price, Juan José Alava

**Affiliations:** 1 Ocean Pollution Research Unit, Institute for the Oceans and Fisheries, University of British Columbia, Vancouver, British Columbia Canada; 2 Toxicology and Environmental Sciences, Department of Bioscience, University of Oslo, Oslo, Norway; 3 Animal Health Centre, British Columbia Ministry of Agriculture, Food and Fisheries, Abbotsford, British Columbia Canada; 4 Fisheries and Oceans Canada (DFO), Fisheries and Aquaculture Management, Vancouver, British Columbia Canada; 5 Applied Remote Sensing Lab, Department of Geography, McGill University, Montreal, Quebec Canada; 6 School of Resource and Environmental Management, Simon Fraser University, Burnaby, British Columbia Canada

**Keywords:** Ocean sciences, Marine biology, Environmental impact, Environmental monitoring

## Abstract

The northeastern Pacific (NEP) Ocean spans the coast of British Columbia (Canada) and is impacted by anthropogenic activities including oil pipeline developments, maritime fossil fuel tanker traffic, industrial chemical effluents, agricultural and urban emissions in tandem with stormwater and wastewater discharges, and forest wildfires. Such events may expose surrounding marine environments to toxic polycyclic aromatic hydrocarbons (PAHs) and impact critical habitats of threatened killer whales (*Orcinus orca*). We analyzed skeletal muscle and liver samples from stranded Bigg’s killer whales and endangered Southern Resident killer whales (SRKWs) for PAH contamination using LRMS. C3-phenanthrenes/anthracenes (mean: 632 ng/g lw), C4-dibenzothiophenes (mean: 334 ng/g lw), and C4-phenanthrenes/anthracenes (mean: 248 ng/g lw) presented the highest concentrations across all tissue samples. Diagnostic ratios indicated petrogenic-sourced contamination for SRKWs and pyrogenic-sourced burdens for Bigg’s killer whales; differences between ecotypes may be attributed to habitat range, prey selection, and metabolism. A mother-fetus skeletal muscle pair provided evidence of PAH maternal transfer; low molecular weight compounds C3-fluorenes, dibenzothiophene, and naphthalene showed efficient and preferential exposure to the fetus. This indicates in-utero exposure of PAH-contamination to the fetus. Our results show that hydrocarbon-related anthropogenic activities are negatively impacting these top predators; preliminary data found here can be used to improve oil spill and other PAH pollution management and regulation efforts, and inform policy to conserve killer whale habitats in the NEP.

## Introduction

Polycyclic aromatic hydrocarbons (PAHs) are ubiquitous in the ocean and are considered to be contaminants of great concern in the environment^1–3^. Consisting of aromatic hydrocarbons with two or more benzene rings, PAHs have attracted the interest of ecotoxicological science due to their widespread prevalence in the environment, and toxic and pervasive behaviour in marine ecosystems. Although this chemical class is composed of several hundred related compounds, only 14–20 of them have been extensively studied and regulated^2^.

In the ocean, there are four primary categories from which PAHs are derived: fuel (petrogenic), incomplete combustion processes (pyrogenic), organic metabolism (biogenic), and transformation processes occurring in sediments^4^. Petrogenic and pyrogenic sources are mainly anthropogenic, through activities such as oil spills, smoking, traffic emissions and other forms of coal, oil, and wood combustion. Such pollutants can enter the marine environment through continental runoffs from watersheds and atmospheric deposition, and are most concentrated in regions like estuaries that are heavily exposed to industrial effluents and other sources of pollution^5,6^. Oil spills are considered the most concerning exposure event of PAHs in the marine environment. Interestingly, these incidents are correlated with major shipping routes and most commonly occur through “deliberate” spills from ships’ operative discharges^1,7^. Once in the aquatic environment, PAHs with high molecular weight tend to stick to the surface of particles and sink to sediments, while those with low molecular weight remain bioavailable in the water where they can be assimilated by organisms through various exposure routes including inhalation, dermal contact, and ingestion, and cause acute or chronic toxicity^8–10^.

Metabolic processes surrounding PAHs are complex and dependent on bioavailability and organism physiology; vertebrates such as marine mammals have pathways that allow them to metabolise and excrete PAHs^11,12^. Consequently, PAHs do not generally biomagnify throughout the food web, and those contaminants detected in upper trophic level species are likely metabolites and/or representative of recent exposure^9^. The weight of evidence from research has supported PAHs’ carcinogenic, mutagenic, immunosuppressant, and toxic effects, as well as their ability to disrupt endocrine systems in marine mammals^2,10,13–16^. Certain derivative compounds of metabolic reactions like benzo[a]pyrene diol epoxide (BPDE) can bind to DNA, causing carcinogenic and/or mutagenic effects. For example, beluga whales (*Delphinapterus leucas*) in the St. Lawrence estuary (LEC), Canada, have had high levels of gastrointestinal cancers and increased incidence of intestinal PAH-DNA adducts that were linked causally with cancer development^17,18^. Takeshita et al*.*^10^ documented toxic effects and health impairments of hydrocarbon exposure (as a function of oil type, concentration, duration and exposure route) in marine mammals, including low reproductive success and fetal distress, impaired stress response (e.g., potential hypothalamus–pituitary–adrenal axis-HPA dsyfunction), lung disease (bronchopneumonia), T-cell disfunction, evidence of liver damage, and decreased body mass following the aftermath of the Deepwater Horizon oil spill in the Gulf of Mexico.

The ocean and nearshore waters of the northeastern Pacific (NEP) Ocean off the coast of British Columbia (BC), Canada, provide essential habitat for two killer whale ecotypes: the marine mammal-eating Bigg’s (transient) killer whale and fish-eating resident killer whales, including northern resident killer whales and critically endangered Southern Resident killer whales (SRKW)^19^. The SRKW population has declined since the early 1990s with over 104 individuals to a current status of 73 or 74 individuals. Primary threats to the survival of this ecotype include reduced prey availability and quality (i.e. of its main prey, Chinook salmon), anthropogenic and physical disturbances (i.e. maritime traffic, acoustic pollution), and chemical pollutants^20–24^. Pollution risk management and assessments, along with pollutant source control programs, are paramount to the protection and conservation of this species. The Canadian *Species at Risk Act* (SARA) protects species at risk from being killed or harmed and protects their critical habitat from degradation or destruction^20^. According to the Recovery Strategy or Action Plan for the SRKW, critical habitat is defined as the habitat necessary for survival and recovery of the listed wildlife species at risk. One of the major objectives (Objective 2) of the SRKW recovery strategy is to “*ensure that chemical and biological pollutants do not prevent the recovery of resident killer whale populations”*^20^.

Coastal waters of BC are impacted by a number of anthropogenic activities such as shipping, transportation, and industrial and urban effluents, and include large industrial hubs like Vancouver and Victoria (Vancouver Island) in the Strait of Georgia. Ongoing oil pipeline development and maritime oil tanker traffic heighten concerns with respect to PAH spills and risk of exposure to killer whales in BC marine waters^25,26^. For instance, a recent report claims ships off the coast of BC discharged 35 million tons of washwater (i.e. from exhaust gas cleaning systems) containing PAHs into the ocean in 2017^27^. An estimated 10% of this discharge was released into SRKW habitat. Additionally, petroleum leaks were reported from two vessels in the NEP. The first shipwreck sank in Nootka Sound, BC, in January 1968 and started to leak dense crude oil in January 2021 (P. Cottrell, pers. comm), and the second event occurred in August 2022 and involved a sunken commercial fishing vessel in Washington State that leaked a combination of diesel fuel, hydraulic fluid, and lubricant oil near San Juan Island^28^. The lack of baseline information on the prior exposure of SRKWs to PAHs and potential contributions of these compounds to morbidity or mortality may have hampered interpretation of any animals that had stranded. Additionally, it is estimated that approximately 2000 tons of PAHs per year is released through forest fires in Canada and approximately 975 tonnes of PAHs are released annually through aluminum smelters—the greatest anthropogenic source of PAHs in Canada^29^. Globally, Asian countries have contributed up to half of the total PAH emissions worldwide via long-range atmospheric transport, with Africa and Indian designated as other large contributors^30,31^.

Free-ranging killer whales are long lived, apex predators, and have large amounts of lipid reserves (fat tissue/blubber) and are, therefore, considered at higher risk to organic pollutant exposure; it is known that these marine mammal species are among the most contaminated cetaceans in the world, exhibiting high concentrations of polychlorinated biphenyls (PCBs)^23,32^. To the best of our knowledge, no research has been performed regarding PAH contamination in tissues, including skeletal muscle and liver samples, of stranded killer whales off the coast of BC. Given this lack of data, the first aim of this study is to establish information on PAH levels in NEP killer whales. This was done by conducting the first assessment of PAH contamination in skeletal muscle and liver samples collected from stranded SRKWs and Biggs killer whales in BC from 2006 to 2018 (Figure S1). The second aim is to determine pollution sources of PAHs in killer whale samples using diagnostic ratios, and the third to investigate maternal transfer of PAH compounds in SRKWs.

## Results and discussion

### Polycyclic aromatic hydrocarbon (PAH) contamination

**Figure 1:**
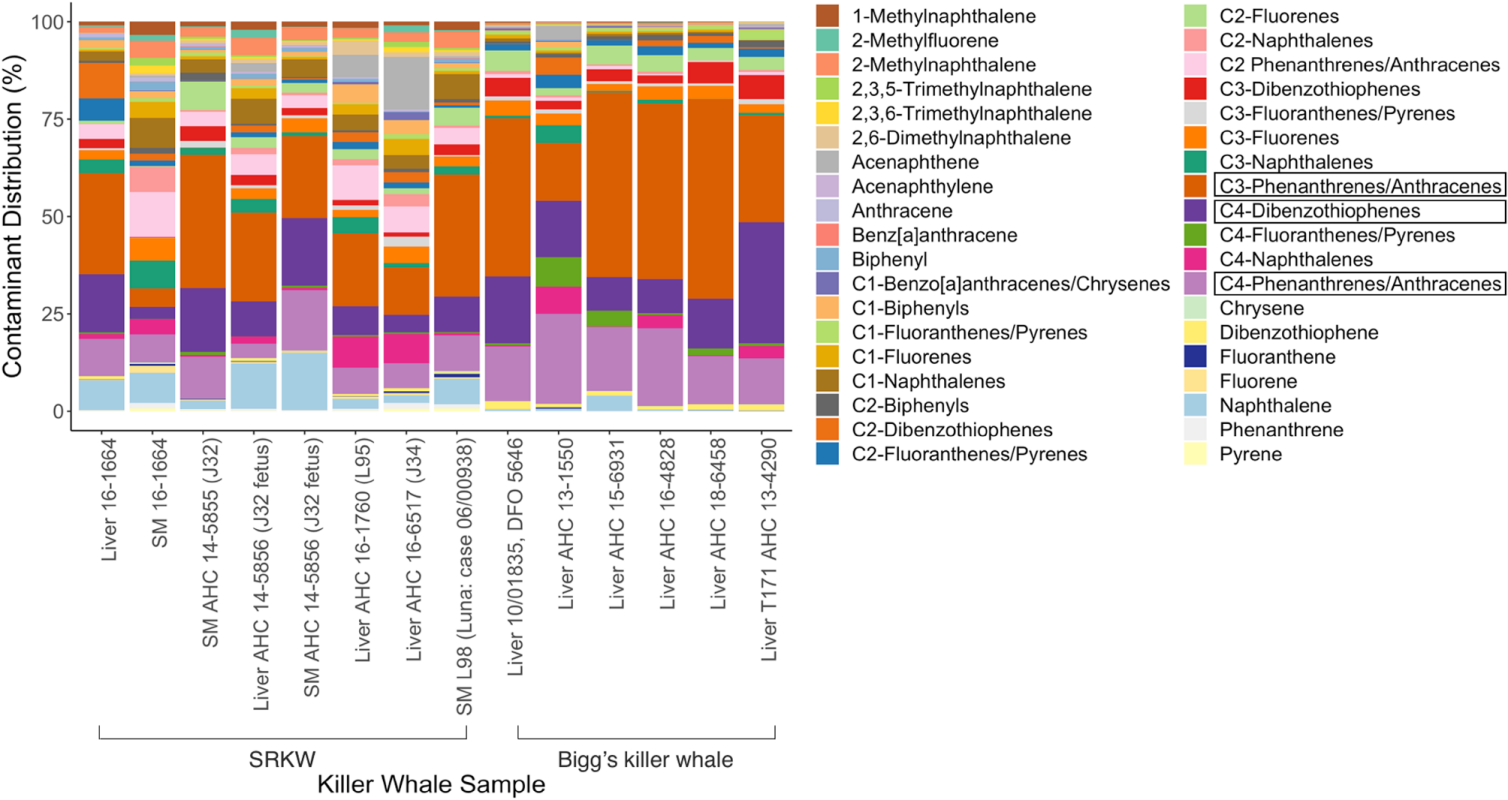
Summary of polycyclic aromatic hydrocarbons (PAHs, lipid weight) distribution pattern (%) for Southern Resident killer whale (SRKW; *O.orca*) and Bigg’s killer whale liver and skeletal muscle (SM) samples (n = 14). The top three most prevalent PAH contaminants are bordered in black.

Of 76 PAHs tested, 38 were detected in at least 50% of samples and included in analysis. Average lipid weight (lw) PAH concentrations across all samples ranged from 1.1 ± 0.28 ng/g lw (acenaphthylene,) to 632.12 ± 192.95 ng/g lw (C3-phenanthrenes/anthracenes; Table S1). Accounting for 33.48% of the total contamination across all samples was C3-phenanthrenes/anthracenes followed by C4-dibenzothiophenes (17.7%) and C4-phenanthrenes/anthracenes (13.1%; Fig. 1); these three compounds were detected at the highest concentrations and were prevalent in all samples. Individual remaining PAH contaminants accounted for less than 5% of the total contamination across all samples.

PAH contamination has been widely studied in cetaceans. Compared to those concentrations found in cetaceans from the Gulf of California, our results showed lower PAH contamination^33^. The present study’s average total PAH concentration across each samples was reported at 1888.14 ± 562.36 ng/g lw while Fossi et al*.*^33^ reported average skin biopsy concentrations ranging from 1316 ng/g lw in the common bottlenose dolphin (*Tursiops truncatus*) to 32,940.6 ng/g lw in female sperm whales (*Physeter macrocephalus*)^33,34^. Over 65% of the reviewed cetacean studies in Fossi et al*.*^33^ had PAH concentrations over 10,000 ng/g lw. The present study had an average PAH concentration that was almost two times higher than that found in blood samples of captive killer whales (1023.2 ± 84.9 ng/g lw) originating from Icelandic waters^13^. Interestingly, there was a discrepancy in high molecular weight (HMW, compounds with four or more rings) and low molecular weight (LMW, compounds with less than four rings) concentrations between these captive killer whales and free ranging killer whales samples analyzed in this case series; average HMW concentrations in captive killer whales (432.4 ng/g lw) were much higher than those found in this study (7.3 ng/g lw) while LMW in captive killer whales (560 ng/g lw) were lower than those found here (830.7 ng/g lw; see Table S2 for molecular weight distinctions (HMW vs LMW) for each PAH contaminant in the present study). These data indicates that captive killer whales are more exposed to pyrogenic PAH sources, reflected by the dominant occurrence of HMW PAHs, as opposed to petrogenic sources indicated by the lower presence of LMW PAHs. In an analysis of SRKW scat samples for PAHs between 2010 and 2013^35^, all samples were less than 10 ng/g wet weight (ww), which was much lower than the average tissue concentrations found in this study (139.4 ng/g ww; Table S1). Considering differences in exposure and metabolic process between, for example, liver and blood, discrepencies may arise when comparing PAH prevelance in different tissue types. However, it is also important to consider that such studies analyzing for PAHs in cetaceans, particularly killer whales, is scarce. Additional studies are warranted for complete comparisons.

Factors such as localized PAH contamination with associated pollution sources in nearby habitat, feeding preferences/prey specialization, individual and species-specific metabolic proceses and capabilities, and state of carcass decomposition (bacterial putrefaction) can play a role in PAH contamination loads and composition in samples^10,12,36,37^. This is the first study to assess PAH contaminant prevalence in skeletal muscle and liver samples of SRKWs and Biggs killer whales of the NEP.

### PAH ratios and source identification

**Figure 2:**
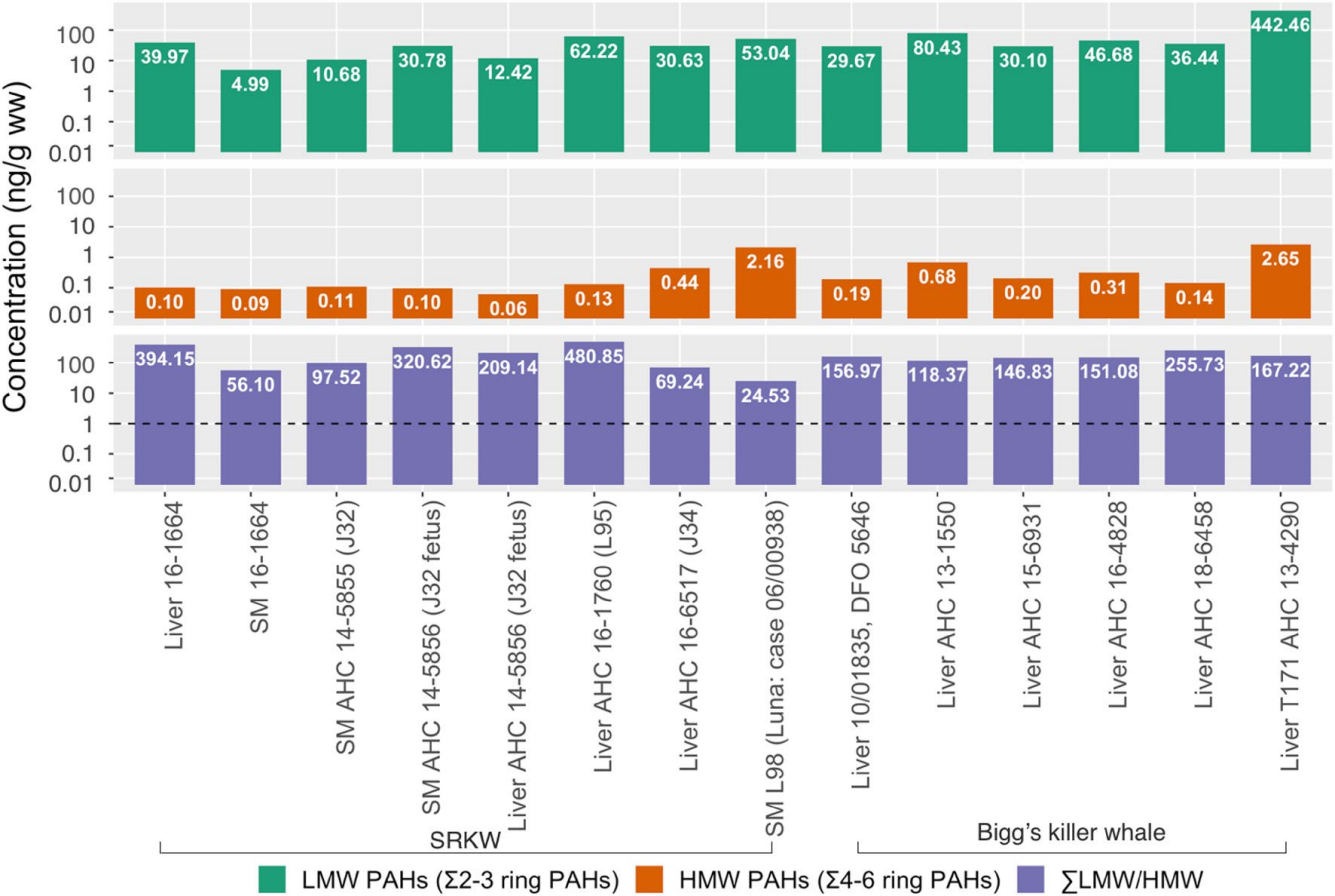
Log-scale distribution of low molecular weight (LMW; 2–3 rings) polycyclic aromatic hydrocarbons (PAHs), high molecular weight (HMW; 4–6 rings) PAHs, and the ratio of ∑LMW/HMW for liver or skeletal muscle (SM) samples (n = 14) of stranded Bigg’s killer whales (*O. orca)* and Southern Resident killer whales (SRKW) reported in ng/g wet weight (ww). The dashed line indicates where those ∑LMW/HMW ratios equal one, ∑LMW/HMW < 1 indicates a pyrogenic source while ∑LMW/HMW > 1 indicates a petrogenic source. **∑**LMW: sum of 1-methylnaphthalene, 2-methylfluorene, 2-methylnaphthalene, 2,3,5-trimethylnaphthalene, 2,3,6-trimethylnaphthalene, 2,6-dimethylnaphthalene, acenaphthene, acenaphthylene, anthracene, biphenyl, C1-biphenyls, C1-fluorenes, C2-dibenzothiophenes, C2-fluorenes, C2-naphthalenes, C3-dibenzothiophenes, C3-fluorenes, C3-naphthalenes, C4-dibenzothiophenes, C4-naphthalenes, dibenzothiophene, fluorene, naphthalene, phenanthrene. ∑HMW: sum of benz[a]anthracene, chrysene, fluoranthene, pyrene.

To control PAH emissions and effectively mitigate hydrocarbon contamination in ocean-coastal environments, it is of paramount importance to recognize sources emitting PAHs^38,39^. Pyrogenic-sourced PAHs are generated in high-temperature events such as incomplete carbon, wood or biomass combustion, and fossil fuel burning. High molecular weight (HMW) PAHs with 4–6 benzene rings (i.e., fluoranthene, pyrene, benz[a]anthracene, chrysene) are used to identify these sources. Conversely, petrogenic PAHs include low molecular weight (LMW) PAHs with 3 or fewer aromatic rings (i.e., naphthalene, acenaphthene, acenaphthylene, fluorene, phenanthrene, and anthracene) that are routinely found in crude oil spills and refined oil^40,41^. PAH concentrations in the studied tissues constitute key matrices to infer whether these killer whales’ PAH exposure are of natural or anthropogenic causes from petrogenic or pyrogenic sources in the marine regions of BC^9,42^. To determine PAH sources, the cumulative sum of LMW (∑LMW; n = 34) compounds, HMW (∑HMW; n = 4) compounds, and the ratio of the two (∑LMW/HMW) was calculated for each killer whale sample. The ∑LMW/HMW was above one in all analyzed samples (∑LMW/HMW > 1; Fig. 2 and Table 1) which indicates a dominant petrogenic source of contamination in killer whales of the NEP.

**Table 1:** Concentrations of polycyclic aromatic hydrocarbon (PAH) contaminants (in ng/g wet weight and lipid weight) and ratios of low molecular weight versus high molecular weight (∑LMW/HMW) PAH compounds in liver and skeletal muscle (SM) samples of stranded Bigg’s killer whales (*O. orca*) and Southern Resident killer whales (SRKW) from British Columbia, Canada (2006–2018).

Killer whale ID	Ecotype	Lipid weight (lw)				Wet Weight (ww)				∑LMW/HMW
		Total PAH concentration (ng/g lw)	Concentration range (ng/g lw)	LMW PAHs (Σ2—3 ring PAHs)	HMW PAHs (Σ4—6 ring PAHs)	Total PAH concentration (ng/g ww)	Concentration range (ng/g ww)	LMW PAHs (Σ2–3 ring PAHs)	HMW PAHs (Σ4–6 ring PAHs)	
10/01,835, DFO 5646 Liver	Bigg’s	2252.4	0.024–915.1	812.8	5.2	82.2	0.0086–33.4	29.7	0.2	157.0
16-1664 Liver	SRKW	400.3	0.018–104.3	245.2	0.6	65.2	0.0029–17	40.0	0.1	394.2
16–1664 SM	SRKW	458.2	0.22–52.8	383.6	6.8	6.0	0.0029–0.69	5.0	0.1	56.1
AHC 13-1550 Liver	Bigg’s	1217.6	0.034–282.1	600.2	5.1	163.2	0.0045–37.8	81.7	0.7	118.4
AHC 14-5855 (J32) SM	SRKW	924.2	0.16–315.9	386.9	4.0	25.5	0.0045–8.7	10.7	0.1	97.5
AHC 14-5856 (J32 fetus) Liver	SRKW	405.2	0.065–92.6	277.3	1.3	18.2	0.0029–4.2	12.4	0.1	209.1
AHC 14-5856 (J32 fetus) SM	SRKW	882.0	0.16–184.7	521.7	1.6	52.0	0.009–10.9	30.8	0.1	320.6
AHC 15-6931 Liver	Bigg’s	3288.9	0.26–1561.4	785.9	5.4	126.0	0.01–59.8	30.1	0.2	146.8
AHC 16-1760 (L95) Liver	SRKW	798.7	0.026–148.7	550.6	1.1	90.3	0.0029–16.8	62.2	0.1	480.8
AHC 16-4828 Liver	Bigg’s	1856.5	0.092–837.6	498.8	3.3	173.8	0.0086–78.4	46.7	0.3	151.1
AHC 16-6517 (J34) Liver	SRKW	986.4	0.067–133.8	709.1	10.2	42.6	0.0029–5.8	30.6	0.4	69.2
AHC 18-6458 Liver	Bigg’s	3182.5	0.11–1631.3	920.2	3.6	126.0	0.0045–64.6	36.4	0.1	255.7
L98 (Luna: case 06/00,938) SM	SRKW	1361.2	1.12–427.8	683.6	27.9	105.6	0.087–33.2	53.0	2.2	24.5
T171 AHC 13-4290 Liver	Bigg’s	8419.7	0.096–2625	4254.4	25.4	875.6	0.01–273	442.5	2.6	167.2

*LMW* low molecular weight and *HMW* high molecular weight.

To further explore PAH sources in killer whales stranded in the NEP, diagnostic ratios (DRs) for each killer whale sample were investigated. PAH DRs are frequently used to distinguish between pyrogenic and petrogenic sources, as well as specific fuels from which PAHs in an environmental compartment are derived^38,43,44^. DRs are predicated on the assumption that PAH isomers have similar physical–chemical properties and that their transformation and degradation in the environment appear at the same rate, thus maintaining the relationship that exists during emission, sample collection, and analysis^38,45,46^. Ant/(Ant + Phe) ratio is commonly used to distinguish petroleum from combustion sources (Table 2, see also ratio contaminant abbreviations); Ant/(Ant + Phe) ratio less than 0.1 indicates petroleum as a PAH source, whereas an Ant/(Ant + Phe) more than 0.1 indicates combustion (i.e. wood, grass, or coal burning) as a PAH source^47,48^. Flu/(Flu + Pyr) > 0.5 and BaA/(BaA + Chr) > 0.35 are commonly associated with combustion, whereas Flu/(Flu + Pyr) < 0.4 and BaA/(BaA + Chr) < 0.2 are associated with petroleum sources. As a result, these ratios are simple to use and have become an important tool for identifying PAH sources, as evidenced by their use in a number of previous studies^44,49–51^.

**Table 2:** Literature-derived diagnostic ratios used for polycyclic aromatic hydrocarbons (PAHs) to assess and infer pollution sources^38,42–44^ using PAH concentrations detected in liver and skeletal muscle of Southern Resident killer whales (SRKWs; *O. orca*) and Bigg’s killer whales stranded in British Columbia, Canada.

PAHs	Diagnostic Ratio	Sources
∑LMW/HMW	< 1	Pyrogenic
	> 1	Petrogenic
Ant/(Ant + Phe)	< 0.1	Petroleum
	> 0.1	Pyrogenic/Combustion
Flu/(Flu + Pyr)	< 0.4	Petroleum
	0.4–0.5	Liquid fossil fuel combustion
	> 0.4	Coal, wood or grass combustion
BaA/(BaA + Chr)	< 0.2	Petroleum
	0.2–0.35	Coal, wood, or grass combustion
	> 0.35	Pyrogenic/Combustion

*Ant* anthracene, *Phe* phenanthrene, *Flu* fluoranthene, *Pyr* pyrene, *BaA* benz[a]anthracene, *Chr* chrysene.

**Figure 3:**
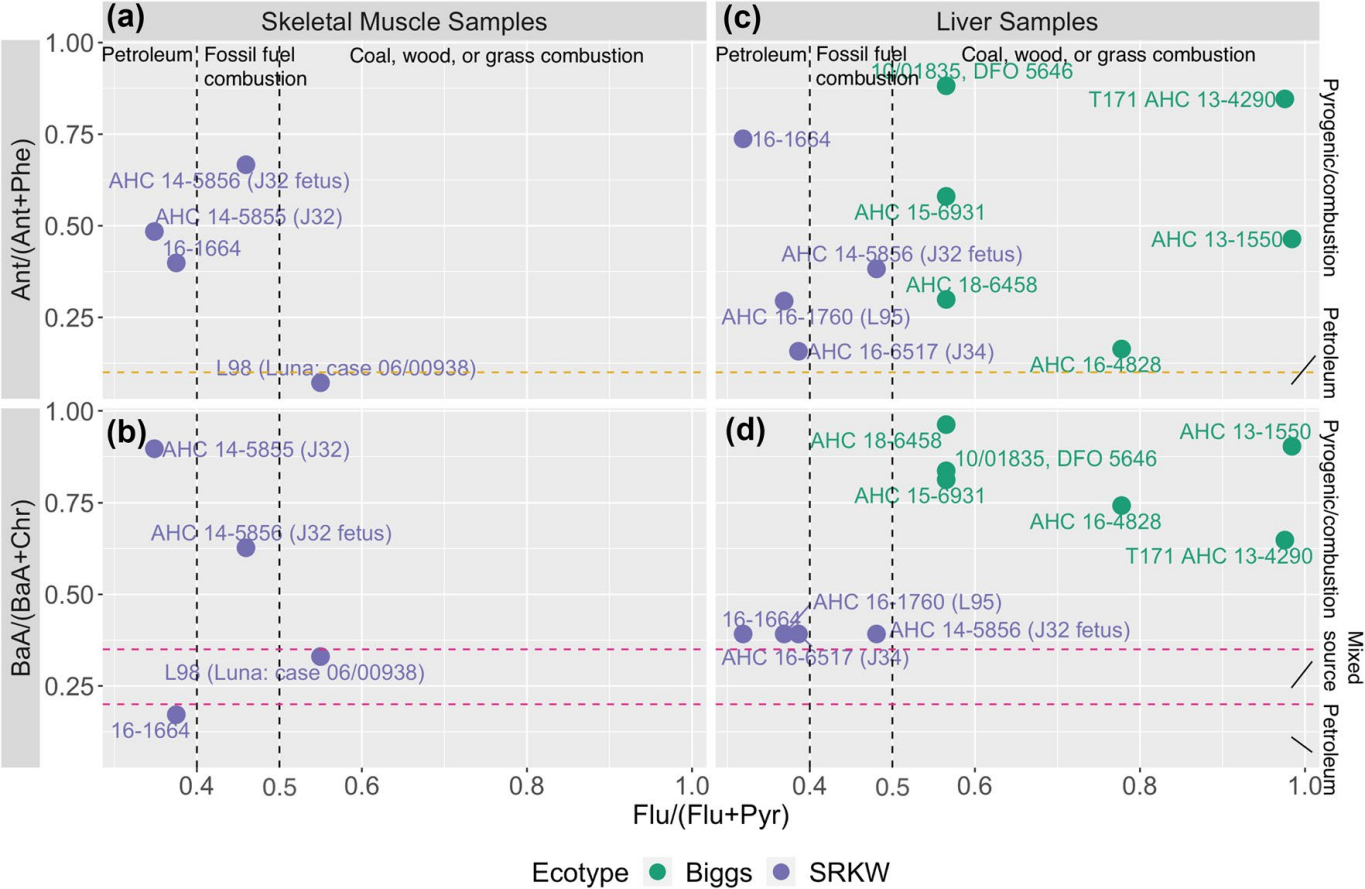
Diagnostic ratio charts based on polycyclic aromatic hydrocarbon (PAH) concentration data (wet weight) measured in stranded Southern Resident killer whales (SRKW; *O. orca*) and Bigg’s killer whales. (**a**) Flu/(Flu + Pyr) and Ant/(Ant + Phe) for skeletal muscle samples, (**b**) Flu/(Flu + Pyr) and BaA/(BaA + Chr) for skeletal muscle samples, **(c)** Flu/(Flu + Pyr) and Ant/(Ant + Phe) for liver samples, (**d**) Flu/(Flu + Pyr) and BaA/(BaA + Chr) for liver samples. Note: *Ant* anthracene, *Phe* phenanthrene, *Flu* fluoranthene, *Pyr* pyrene, *BaA* benz[a]anthracene, *Chr* chrysene. See Table S3 for diagnostic ratio values.

Diagnostic ratios of skeletal muscle samples collected from SRKWs (Table S3) identify petroleum and liquid fossil fuel combustion to be the predominant source of PAH contamination. Data presented in Fig. 3a show ratios of Ant/(Ant + Phe) > 0.1 for all samples, with the exception of L98. This indicated that petroleum combustion activities may be responsible for PAH contamination in skeletal muscle samples. A Flu/(Flu + Pyr) < 0.5 in skeletal muscle samples (Fig. 3a,b) suggested liquid fossil fuel combustion (vehicle and crude oil) as a source of PAHs in the case of J32 fetus (0.4 < Flu/(Flu + Pyr) > 0.5) and petroleum combustion to be the sources for all other samples^38,47^. Contrarily, a Flu/(Flu + Pyr) > 0.5 for L98 indicated that PAH contamination originated from coal/wood/grass or petroleum combustion. While still considering a ratio of Flu/(Flu + Pyr) < 0.5, BaA/(BaA + Chr) DRs were reported above 0.35 for two samples (J32 and J32 fetus) suggesting petroleum combustion sources of PAHs in these individuals (Fig. 3b). Detection of 0.2 < BaA/(BaA + Chr) > 0.35 in tissue samples from L98 (Fig. 3b) further substantiated L98 as having PAHs contaminant sources from coal, wood, or grass combustion, while sample 16-1664 showed petroleum based PAH contamination (Flu/(Flu + Pyr) < 0.4 and BaA/(BaA + Chr) < 0.2; Fig. 3b).

The SRKW L98, also known as “Luna”, was born in Puget Sound (Washington State, US), separated from his mother while still young, and spent 5 years in Nootka Sound, an ocean inlet of western Vancouver Island (BC, Canada), where he had extensive human interaction. His variable habitats and close contact with humans, urbanization, and industries may have contributed to his combined fingerprint of PAH sources.

Overall, liver samples of Bigg’s killer whales had a predominantly pyrogenic PAH profile whereas in SRKWs, PAHs were primarily sourced from petrogenic origins. All liver samples reported Ant/(Ant + Phe) values more than 0.1, indicating pyrogenic origin (Fig. 3c, top right). Bigg’s killer whale liver samples reported DRs of Flu/(Flu + Pyr) > 0.5 and BaA/(BaA + Chr) > 0.35, further indicating these samples are contaminated with pyrogenic source (coal, wood, or grass combustion) of PAHs (Fig. 3d). SRKWs showed Flu/(Flu + Pyr) < 0.5, with J32 fetus PAH contamination being sourced from liquid fossil fuel combustion with the remaining three being petroleum-combustion sourced. This further supports that highly mobile Bigg’s killer whales are more contaminated with PAHs derived from coal, wood, or grass combustion whereas more urban SRKWs are contaminated with PAHs derived from petroleum or petrogenic combustion. These PAH ratio patterns are consistent with the PAH ratios observed in suspended particles and sediments from the Fraser River Basin system from BC, Canada^38^. PAH ratios and total concentration data from sediments samples revealed a basin lightly impacted by different sources in remote locations, especially near roads, but heavily impacted in urban areas, particularly near Vancouver^38^. Yunker et al*.*^38^ reported that PAH contamination sources shift from biomass (e.g. wood and grass) burning in remote areas to vehicle emissions in urban locations, where stormwater and wastewater discharges appear to collect PAHs from urban environments and release them as point sources.

Liver analysis demonstrated differences in PAH levels and origins in the two killer whale ecotypes in the NEP. A principal component analysis (PCA) was performed on the matrix of PAH concentrations detected in liver samples to further differentiate PAH sources between SRKWs and Bigg’s killer whales (skeletal muscle samples were not available for Bigg’s killer whales, thus these samples were not included in the PCA assessment). The first two principal components (i.e. PC1 and PC2) accounted for 46.3% and 29.6% of the total variation in hydrocarbon contaminant loads (i.e. most HMW-PAHs and LMW-PAHs were clustered for Bigg’s killer whales versus loads primarily dominated by LMW-PAHs grouped for SRKWs), respectively (see Figure S2). A redundancy analysis (RDA) found that ecotype provided a significant explanation of the variation in the contaminant data set (39.2%, *p* = 0.003, *F-score* = 6.26), and a significantly higher LMW PAH concentration was found in Bigg’s killer whales compared to SRKWs (Welch Two Sample t-test, *p* = 0.003). This supports that ecotype influences PAH contamination and further suggests that SRKWs exhibited a more dominant petrogenic profile, while Bigg’s killer whales had a predominately pyrogenic PAH profile. HMW PAHs showed no statistically significant difference in concentration between ecotypes (Welch Two Sample t-test, *p* > 0.05), which may be due to the limited number of HMW compounds (n = 4) analyzed in the present study.

In summary, PAH isomer ratios indicated an overall petrogenic source of PAH contamination in all killer whale samples (∑LMW/HMW > 1), and DRs of individual samples gave insight to more specific PAH sources. SRKW skeletal muscles samples showed DRs that supported the results of the isomer ratios: PAH contamination was derived from petrogenic or petrogenic-combustion sources, with the exception of L98. Liver sample DRs distinguished interesting differences in PAH contamination between Southern Resident and Bigg’s killer whale ecotypes; PAHs found in Bigg’s killer whales were derived from pyrogenic sources, while those found in SRKWs arose from petrogenic sources. Trends in SRWKs may be attributed to their habitat as this ecotype remains seasonally inshore and nearby coastal waters (Strait of Georgia, Johnstone Strait, and Burrard Inlet, BC), which surrounds industrial, urban and residential areas like the greater metropolitan of Vancouver and Victoria. Here, materials such as coal and oil are loaded onto ships at Vancouver ports and transported, increasing the likelihood of oil spills and ship wastewater discharge^52^. Additionally, an abundance of stormwater and urban wastewater can collect PAHs and release them into the marine environment^38^. Other sources may include vehicle exhaust, emissions from oil-fueled ships, and other industrial emissions^38^. Interestingly, PAH concentrations have been found to be above regulation limits in the sediments of Burrard Inlet, BC, further suggesting PAH exposure to infauna organisms and those transiting these waters, such as the SRKW^36, 53^. High concentrations of PAHs have also been found in sediments surrounding Kitimat, BC, where an aluminum smelter is situated; research has suggested that PAH air emissions from such smelters can be deposited within an 80 km radius and dispersed up to 30 km in aqueous media^52^. A naturally-occurring source of pyrogenic PAHs, particularly along the west coast of Canada, are forest wildfires with prevailing winds facilitating long-range atmospheric transport of PAH compounds to Bigg’s killer whale more remote habitats^52^. Further PAH-source analyses using additional DRs including An/178 and BaA/228 suggested diesel oil, Australian crude oils and fluid inclusion oils, and coal for petroleum sources, and wood, grasses, diesel, No.2 fuel oil, and bituminous coal for combustion sources are common reoccurring sources in the killer whale samples^38,54^.

### Maternal transfer of pollutants

**Figure 4:**
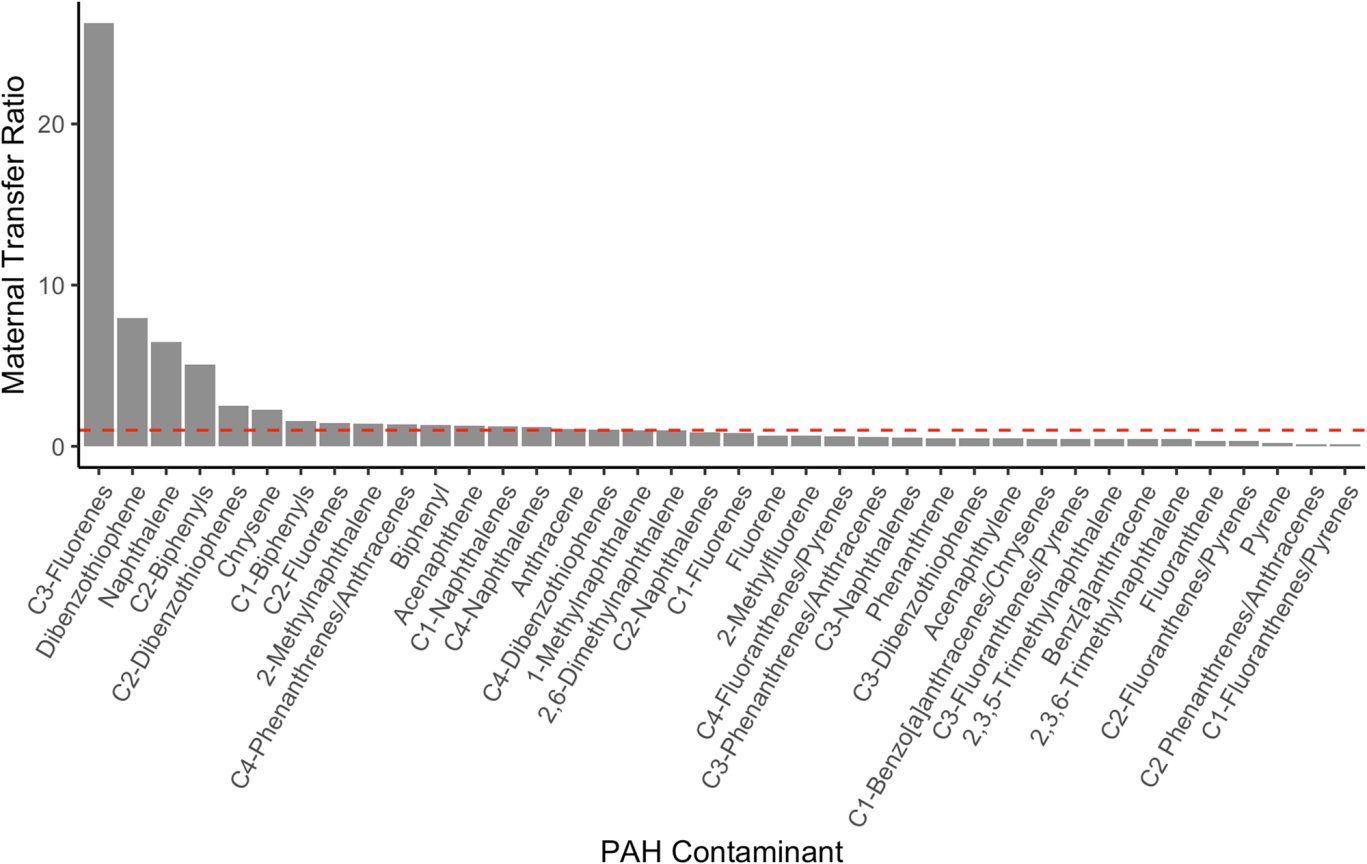
In-utero maternal transfer ratios (MTR) in J32 Mother and J32 Fetus Southern Resident killer whales (SRKW; *O. orca*). MTRs calculated based on skeletal muscle (SM) polycyclic aromatic hydrocarbon (PAH) concentrations (ng/g lipid weight) in AHC 14–5855 (J32) (J32 Mother) and her calf, AHC 14-5856 (J32 Fetus). MTR > 1 indicate the fetus was efficiently and preferentially exposed to the given contaminant while contaminants with MTR < 1 indicates they were not. The dashed red line indicates MTR = 1, representing equal partitioning of contaminant concentrations between fetus and mother.

Our results support maternal transfer of PAHs to offspring. Given the skeletal muscle (SM) samples of a mother-fetus pair (i.e., J32 Mother and J32 Fetus), we had the opportunity to investigate in-utero maternal transfer of PAHs by calculating maternal transfer ratios (MTR). Our data show efficient and preferential contaminant exposure (MTR > 1) to the fetus in 16 PAH compounds, with the three highest ratios being C3-fluorenes (26.3), dibenzothiophene (8.0), and naphthalene (6.5; Fig. 4 and Table S2). Over threefold larger than dibenzothiophene, C3-fluorenes showed the highest MTR, with fetus and mother C3-fluorene burdens reported at 31.7 ng/g lw and 1.2 ng/g lw, respectively. Similar trends were observed in maternal transfer rates as C3-fluorenes was reported at 98.3%, dibenzothiophene at 94.4% and naphthalene at 93.3% (Table S2).

**Figure 5:**
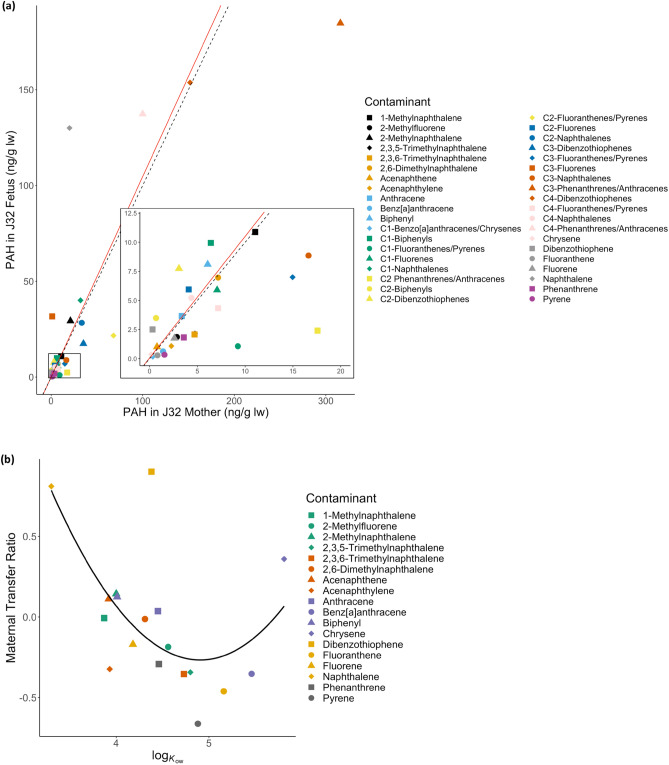
Maternal transfer analyses of PAHs in skeletal muscle of AHC 14–5855 (J32 Mother) and AHC 14–5856 (J32 Fetus) Southern Resident killer whale (SRKW; *O.orca*). **(a)** Relationship (red line; *p* = 1.38 × 10^−08^, r = 0.77, slope = 1.05) between PAH concentrations (n = 38) in each sample. The dashed line indicates a 1:1 concentration ratio, contaminants above this line indicate a higher contaminant concentration in J32 Fetus compared to J32 Mother. **(b)** Relationship (best fit quadratic curve) between the octanol–water partition coefficient (log K_ow_) and maternal transfer ratio (MTR) of each contaminant (n = 18) derived from each sample. Note: this relationship is presented in log scale. Please refer to Table S2 for contaminants’ log K_ow_ values.

A significant positive correlation (Spearman Rank Correlation; *p* = 1.38 × 10^−08^, *r* = 0.77) was found between contaminant concentrations in the mother-fetus pair (Fig. 5a). These findings suggest maternal transfer is an exposure pathway of PAH contaminant to killer whale fetuses, and provides evidence to maternal offloading of PAHs. It also suggests fetuses may have the inability to sufficiently biotransform and detoxify these contaminants via metabolic pathways. This is consistent with previous research that shows mammalian fetal systems are not equipped with mature detoxification systems^55–57^. Although previously studied in fish and reptile species and humans, little is known about maternal transfer of PAHs in marine mammals, especially killer whales^58–60^. Efforts to conduct further analysis on fetuses or neonates that succumb are warranted.

The relationship between maternal transfer ratios and octanol–water partition coefficient (K_*ow*_) was explored to better understand the transfer of contaminants from J32 Mother to J32 Fetus. Because PAHs must travel through hydrophilic and hydrophobic environments within the mammal, a compound’s lipophilic nature and biochemical structure can affect absorption and distribution^61^. The K_*ow*_ is a common bioaccumulation metric to express the lipophilicity of a compound as the octanol–water solution mimics the hydrophobic bilayer. K_*ow*_ values change based on compound size; log K_*ow*_ increases from approximately three to 10 from 2-ring PAHs to 10-ring PAHs^62^.

Data presented in Fig. 5b suggests a negative trend between maternal transfer ratio and log K_*ow*_; PAH compounds with LMW and lower log K_*ow*_ may be more readily transferred across placental membranes compared to contaminants with HMW and higher log K_*ow*_. For example, naphthalene is a compound with two rings (LMW), a low log K_*ow*_, and a high MTR, whereas benz[a]anthracene has four rings (HMW), a high log K_*ow*_, and a low MTR (Fig. 5b and Table S2). According to these results, compounds most transferred to the fetus (i.e. higher MTR) are primarily LMW compounds and, thus, considered more easily absorbed. These findings are consistent with previous research in both marine mammals and humans reporting selective transfer potential of LMW PAHs and PAHs with lower K_*ow*_ from mother to fetus^37,63–65^.

### PAH metabolism implications

As PAHs undergo metabolism or biotransformation via CYP1A cytochrome P450 enzymes in the aquatic food web^66,67^, tissue levels likely reflect only recent PAH exposure^13,68,69^. However, persistence of PAH contaminants may occur in those species chronically exposed to PAH pollution^70^. Harris et al*.*^9^ reported that while the depuration (metabolization or excretion) of parent PAHs was observed in sea otters from British Columbia, the biomagnification of alkyl PAHs was present in this marine mammal species predating on low trophic level macrobenthic biota. This biotransformation process may discount biomagnification of parent PAHs throughout trophic levels, although different food web composition and feeding ecology may well be impacting the overall PAH contamination and accumulation in tissues of SRKWs and Bigg’s killer whales. For example, SRKWs (fish-eaters) may have lower PAH contamination compared to Bigg’s (mammal-eaters) (Figure S3) as studies have considered fish to have higher PAH metabolism efficiency^71^, while pinnipeds of the Bering Strait have been found to have similar PAH contaminant levels to those of killer whales in the present study^72^. To the best of our knowledge, specific PAH metabolic capabilities of killer whales are unknown. Despite the PAH metabolism capacity in marine mammals, this is the first study to present source identification of PAHs in high trophic-level cetacean species^12,73^. These findings indicate that anthropogenic influences, including oil spills, petroleum waste and combustion activities from industries, among other processes, are observed in even the top marine predator of the NEP.

### Confounding variables and study limitations

Relationships between PAH contamination, lipid content, body condition indices (BCI), carcass condition codes, age, sex, and ecotype (see Table S4 for killer whale sample biometric data) associated with each sample were investigated as confounding variables in the contaminant analyses. Significant correlations were found between percent (%) lipid and PAH concentration, and between BCI and PAH concentration. Percent (%) lipid was not correlated with any samples’ BCI and carcass condition codes. Furthermore, BCI and carcass condition codes were not significantly correlated. For a summary of significant correlations and differences between confounding variables and PAH concentrations, please refer to the Supplementary Information (Table S5).

No significant correlation was found between age and total contamination in each sample, indicating age did not impact PAH contamination in these samples. However, significant differences in contamination levels were found between males (n = 5) and females (n = 6); all contaminants with significant concentration differences were higher in males. There is limited evidence of such toxicokinetic process occurring for PAHs in marine mammals, potentially due to metabolism of these contaminants as previously discussed; however, this trend has been observed in lipophilic compounds in which female killer whales offload their contaminants to their offspring via the placenta and milk, resulting in lower contaminant burdens^24,32,74^.

Concentrations of PAHs in SRKW (n = 3) versus Bigg’s (n = 4) killer whale calves (calf cohort includes neonates and fetus) were explored; two contaminants were significantly higher in SRKW calves while 13 were significantly higher in Bigg’s killer whale calves (Figure S3). Similar to previous discussions, these differences may be attributed to habitat^23^; whereby SRKWs frequent inshore, coastal areas, Bigg’s killer whales frequent broader outer-coastal and inshore regions^23,32,75^. Additionally, SRKW have a preference for fish, particularly Chinook salmon, in contrast to the predominantly mammalian diet of Bigg’s whales.

While considering the confounding variables presented here, there are additional limitations in the present study. First, and considering PAH levels likely reflect only recent exposure, tissue collection date was not considered as a confounding variable in this study as it would have compounded with other confounding variables. For example, from 2006 to 2018, sampling was not consistent (as stranding events are opportunistic) and samples were not distributed evenly in terms of sex, thus making it difficult to determine patterns in PAH contamination over time. Second, it has been reported that metabolic processes dominate the fate of PAH compounds in killer whales^9,13^, which may ultimately impact the PAH concentrations reported. Although carcass condition codes were considered a confounding variable and a tool to infer the degradation of the mammal, and therefore the potential breakdown and metabolism of PAHs, it is difficult to infer the complete extent of these processes. This limitation can be extended to DRs which assume that PAHs transform and degrade in the environment at the same rate, meaning ratios remain constant from time of emission to sample analysis^38,45,46^. This assumption may not be representative of actual processes occurring in the environment. Third, there was a low representation of HMW compared to LMW PAHs when discussing DRs and PAH sources; this presents analytical bias and alters the reliability of PAH-source determination in the study.

It must be highlighted that results presented in this study are based on a small sample size (n = 14). While recognizing that such killer whale samples are opportunistic and logistically difficult to obtain, it presents challenges regarding the confidence of the results’ statistical significance and limits the ability to apply results to a population level. For example, extrapolating evidence of maternal transfer from a single (n = 1) mother-fetus sample pair may not be statistically suitable and may not be representative of maternal transfer processes occurring in other killer whales. Nonetheless, these data are important to present as there is a lack of research pertaining to PAH contamination in marine mammals, specifically Bigg’s killer whales and endangered SRKWs.

## Conclusion

This study provided the first evidence of PAH contamination in skeletal muscle and liver samples of stranded SRKWs and Bigg’s killer whales inhabiting the NEP waters of British Columbia, Canada. C3-phenanthrenes/anthracenes, C4-dibenzothiophenes, and C4-phenanthrenes/anthracenes (LMW compounds) PAH contaminants were most prevalent across all tissue samples. The findings of this study likely represent only recent exposure of PAHs, and the ecotoxicological impacts of these compounds are unknown in these killer whale ecotypes. Notably, this research distinguishes sources of contamination in these tissue samples as PAH burdens were derived primarily from pyrogenic sources in Bigg’s killer whales and from petrogenic sources in SRKWs. Differences between PAH pollution sources identified in these ecotypes may be attributed primarily to habitat range, feeding preferences and behaviour, metabolism, and specific food web composition (i.e., fish-eating SRKW versus marine mammals-eating Bigg’s killer whales). This study is also the first to document in-utero maternal transfer of PAHs from a dam to its fetus in SRKW skeletal muscle samples and, therefore, serves as preliminary information on the maternal transfer of PAHs in marine mammals. Primarily LMW PAH contaminants were efficiently and preferentially exposed to the fetus (MTR > 1), with C3-fluorenes, dibenzothiophene, and naphthalene having the highest MTR and maternal transfer rates.

The presence of PAH contamination in these sentinel marine mammals is an indication of the negative impact of environmental pollutants on ecosystem health; therefore, PAH prevalence in killer whales is important to document and understand. Findings presented here are based on several limitations, including sample size, and should be considered as preliminary results on which future studies with larger sample sizes may provide more robust and statistically significant results. Nonetheless, this study is important as it contributes to the knowledge gap regarding PAH contamination in marine mammals, specifically endangered killer whales inhabiting the NEP. With anthropogenic pollution having the potential to expand in the future and further impact marine ecosystems, this research provides information upon which risk management of hazardous chemical pollutants and regulation-related decisions can be made to conserve and protect SRKW critical habitat and surrounding areas from further chemical pollution.

## Materials and methods

Specific subsections of the MATERIALS AND METHODS, including *ethics declaration*, *sample collection, quality assurance/quality control (QA/QC),* and *data treatment and statistical analysis* are done in accordance to those outlined in Lee et al.^24^ Please refer to this citation if more information is required than that provided below.

### Ethics declaration

The study involved the post mortem examination of dead killer whales under permit from the Department of Fisheries and Oceans Canada (DFO licence number XMMS 2 2021) with no live animal capture or sampling as part of the investigation.

### Sample collection and additional data collection

Between 2006 and 2018 twelve dead whales were reported along the coast of BC (Figure S1; Table S4 includes biometrics and description on each sample). Animals were initially photographed for identification, then morphometrics were compiled and the stranding location, date, sex, ecotype, age, class, carcass condition code and BCI was recorded^24^. Necropsies and collection of tissue specimens were performed on these individuals (excluding L98 [Luna]: case 06/00938) following systematic gross necropsies according to established protocols^24,76^.

### Analytical methods

Tissue analysis of PAH contaminants was conducted at SGS AXYS Analytical Services Ltd (Sydney, BC) following procedure MLA-021, Rev. 12, Ver. 07. Method MLA-021 is an extended version of EPA 8270C/D modified by EPA 1625B and the laboratory is ISO 17,025 accredited with 25 years of experience in water, solids, and tissues. MLA-021 used isotope dilution/recovery correction and internal standard quantification; liver and SM samples were spiked with labelled (deuterated) surrogate standards and were extracted through Soxhlet extraction with dichloromethane. The extracts were cleaned up through column chromatography on silica, and gel permeation column chromatography. The reliable and validated separation through fractionation of the silica column cleanup extract separates alkanes from the PAH fraction. This removes a significant source of background matrix, particularly for PAH alkylated groups (the matrix effect is often seen as Unresolved Complex Matrix or UCM in methods that target hydrocarbon fingerprinting). The extracts were further cleaned using all or some of the following procedures: washing with a base, alumina or biobead column chromatography. The isotope dilution technique increased accuracy by compensating for losses of target analytes based on measured recovery of labelled surrogates, accounted for any bias as a result of matrix variation, and allowed for the more aggressive clean-up resulting in better chromatography with less noise and lower detection limits in field samples. Next, extracts were instrumentally analysed by low-resolution mass spectrometry (LRMS) using an RTX-5 capillary GC column. Two mass fragments were monitored for each of the PAH and specific alkylated PAH target analytes and their associated surrogate standard. They must provide a response within a given ratio to be a valid and considered detected, providing a high degree of positive identification. Quantification is performed from a 5 point calibrated response curve with calibration verification performed at least every 12 h. PAH compounds plus technical mixtures with calibrated alkylated PAH group responses are used in the calibration and quantification of alkylated groups and improve the accuracy of the alkylated group compounds reported versus lesser industry standards. Alkylated groups are calculated from sums of responses for specific ions representing a group over a set period of retention time in one channel. A second channel is also monitored and when a response occurs in this channel, the value of the particular retention time is quantified in both channels with method criteria applied for ratio acceptance of the multiple ions. This is a superior quantitative approach compared to open scan semiquantitative alkylated group “fingerprints” generally used in the industry for alkylated group values.

### Quality assurance/quality control (QA/QC)

The PAH analysis followed the Quality Control Acceptance Criteria of SGS AXYS Analytical Services (method MLA-02,1 Rev. 12, Ver. 07). Briefly, liver and SM tissue samples were analyzed in batches consisting of a maximum of 20 samples, one procedural blank and one spiked matrix (OPR) sample. The batch was carried through the complete analytical process as a unit. For sample data to be reportable, both instrument and batch QC established acceptance criteria must be met, including but not limited to procedural blank levels, OPR recovery acceptance criteria, surrogate recovery criteria, instrument sensitivity, ion abundance, and calibration criteria.

### Data treatment and statistical analysis

Data treatment and statistical analyses were performed using RStudio version 4.0.2. Contaminant concentrations were blank corrected or appropriately substituted according to Lee et al*.*^24^. Sample reporting limits for PAH data ranged from 0.0041 to 0.0663 ng/g wet weight (ww). If more than 50% of samples reported not detected (ND) for a given contaminant, the contaminant was no longer considered in the analysis^9,42^. Each sample was analyzed for 76 contaminants, however, only 38 were evaluated in the present study as half were not detected in over 50% of the samples (refere to Table S6 for all raw data).

Statistical analyses used a significance level of 0.05 (*α* = 0.05). After checking data for normality (Shapiro Wilk test) and homogeneity (Brown-Forsythe test), correlation analyses (Pearson’s Product-Moment correlation or Spearman Rank correlation) were used to examine relationships between PAH concentration in each sample with lipid content, body condition indices (BCI), carcass condition codes, age, sex, and ecotype. For comparisons between confounding variables, the parametric Welch’s Two Sample *t*-test was applied to normally distributed data, while the non-parametric Wilcoxon Rank Sum Exact test was applied to non-normal data.

Carcass condition codes and BCIs were obtained through published and unpublished data^77^. Carcass condition ranges from 1 (alive mammal) to 5 (mummified mammal) based on Geraci & Loundsbury^78^. BCI is a function of the individual killer whale’s girth and length (BCI = girth/length) and ranges from a poor value (BCI = 0.5–0.6), good (BCI = 0.6–0.7), and artificially inflated to mimic bloated or pregnant individuals (0.7–0.8)^77^. BCI data was obtained for only six individuals (Table S4).

Maternal transfer ratios (MTRs) were based on SM samples and were calculated using the following equation for each contaminant: (contaminant concentration in J32 Fetus SM)/(contaminant concentration in J32 Mother SM). Note that liver tissue samples were not available for J32 Mother, thus MTR analyses are SM based. Maternal transfer rates (%) were calculated using the following formula^79^: (contaminant concentration in fetus)/(contaminant concentration in mother + fetus) × 100. Note that, unlike in Gebbink et al*.*^79^, contaminant burdens could not be used in this equation as total SM mass could not be calculated.

### PAHs source identification

To identify potential sources of PAHs, PAH diagnostic ratios were calculated using the wet weight PAH concentrations detected in liver and skeletal muscle samples collected from the stranded killer whales. Source identification data was presented in wet weight as the aim was to understand the putative PAH sources in coastal ecosystem habitats of the NEP to which killer whales were exposed. This is opposed to the internal exposure concentrations which are frequently lipid-normalized due to the influence of lipids on PAH burdens. A PCA and RDA was applied to further explore and identify PAH pollution source pattern and contributions of LMW versus HMW PAHs in liver tissues of the two ecotypes.

### Supplementary Information

Supplementary Information 1.

Supplementary Table S1.

Supplementary Table S6.

## Data Availability

All data is available in the main text or the Supplementary Information.
