# Supplementary material for: Polycyclic aromatic hydrocarbon (PAH) source identification and a maternal transfer case study in threatened killer whales (Orcinus orca) of British Columbia, Canada
Source: Sci Rep. 2023 Dec 19;13:22580. doi: 10.1038/s41598-023-45306-w (PMC10730697; doi:10.1038/s41598-023-45306-w)
Supplement: Supplementary file 1 — Supplementary Information 1. [file 41598_2023_45306_MOESM1_ESM.docx]

**Polycyclic aromatic hydrocarbon (PAH) source identification and a maternal transfer case study in threatened killer whales (*Orcinus orca*) of British Columbia, Canada**

Kiah Lee^1,2,*^, Stephen Raverty^1,﻿3^, Paul Cottrell^4^, Zeinab Zoveidadianpour^1,5^, Brendan Cottrell^6^, Dana Price^1^ & Juan José Alava^1,*^

^1^Ocean Pollution Research Unit, Institute for the Oceans and Fisheries, University of British Columbia, Canada.

^2^Toxicology and Environmental Sciences, Department of Bioscience, University of Oslo, 0371, Oslo, Norway.

^3^Animal Health Centre, BC Ministry of Agriculture and Food, Abbotsford, BC, Canada.

^4^Fisheries and Oceans Canada (DFO), Fisheries and Aquaculture Management, Vancouver, BC, Canada.

^5^Department of Marine Biology, Faculty of Marine Science and Oceanography, Khorramshahr University of Marine Science and Technology, Khuzestan Province, Khorramshahr, C623+QG5, Iran.

^6^Applied Remote Sensing Lab, Department of Geography, McGill University, Quebec, Canada.

^*^Corresponding authors: [kiahlee@student.ubc.ca](mailto:kiahlee@student.ubc.ca); [j.alava@oceans.ubc.ca](mailto:j.alava@oceans.ubc.ca)

**Supplementary Information**

Table of Contents

[Figure S1 S3](#_Toc144925618)

[Figure S2 S4](#_Toc144925619)

[Figure S3. S5](#_Toc144925620)

[Table S1. S6](#_Toc144925621)

[Table S2. S6](#_Toc144925622)

[Table S3. S7](#_Toc144925623)

[Table S4. S9](#_Toc144925624)

[Table S5 S11](#_Toc144925625)

[Table S6. S12](#_Toc144925626)

[References S12](#_Toc144925627)


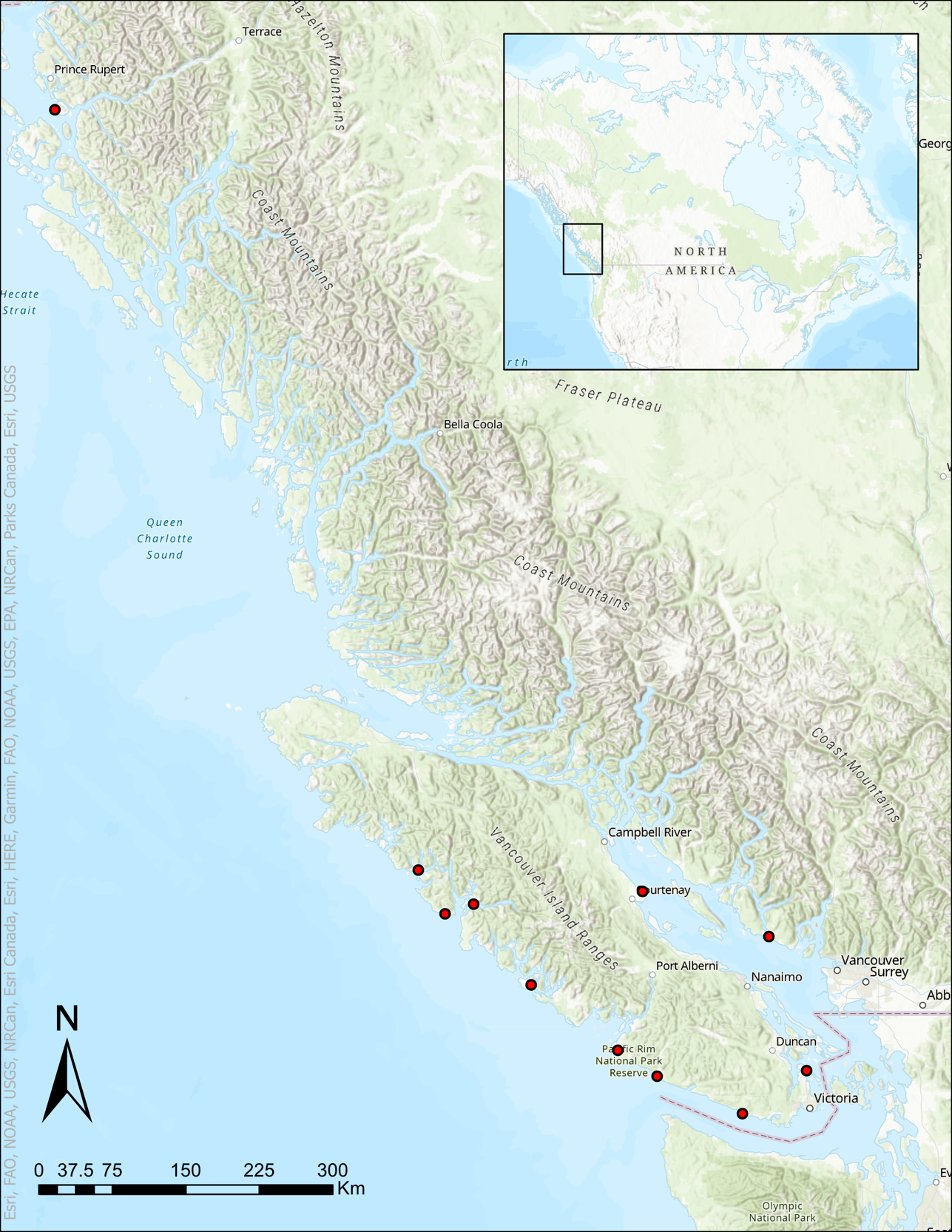


**Figure S1.** Locations of the 12 stranded killer whales (*Orcinus Orca*) analyzed in the present study along the coast of British Columbia, Canada, from 2006 to 2018. Note: two stranding locations are overlapping due to the mother-fetus sample pair (J32 Mother and J32 Fetus) that were collected together. Map was generated using ArcGIS 3.0.2. (<https://www.esri.com>). Reprinted (adapted) with permission from {Lee, K. *et al.* Emerging contaminants and new POPs (PFAS and HBCDD) in endangered Southern Resident and Bigg’s (Transient) killer whales (*Orcinus orca*): *In utero* maternal transfer and pollution management implications. *Environ. Sci. Technol.* (2023) doi:10.1021/acs.est.2c04126}. Copyright {2023} American Chemical Society.


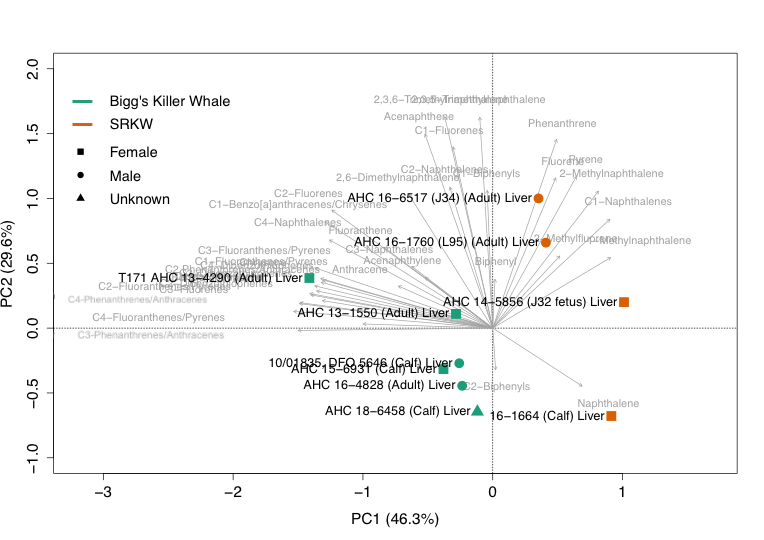


**Figure S2.** Principal Component analysis (PCA) biplot based on polycyclic aromatic hydrocarbon (PAH) contaminant concentrations in liver samples of Southern Resident killer whales (SRKW) and Bigg’s killer whales (*O.* orca, n = 14) stranded in British Columbia, Canada. Response-variable loadings (i.e. PAH contaminants) are depicted as grey arrows, and explanatory variables (i.e. ecotype and sex) are passively plotted.

**Figure S3.** Difference in mean polycyclic aromatic hydrocarbon (PAH) contaminant concentration (ng/g lipid weight) in Southern Resident calves (n=3) and Bigg’s (Transient) killer whale (*O. orca*) calves (n=4). PAH contaminant data set are presented in log scale and standard error bars are shown. Note: * denotes those PAH contaminants significantly higher in Bigg’s killer whale calves, while ** denotes those significantly higher in SRKW calves.

**Table S1.** Wet weight and lipid normalized contaminant concentration (ng/g) for each killer whale (*O. orca*) sample (SAMPLE ID). Data is blank corrected.

See excel sheet Table S1.

**Table S2.** Molecular weight classification **(**L = low molecular weight, H = high molecular weight, NA = not applicable), maternal transfer ratio (MTR) and maternal transfer rate (%) for each contaminant and its associated octanol-water partition coefficient (log *K*_ow_ ).

| **Contaminant** | **Molecular**  **Weight** | **Number of rings** | **MTR** | **Maternal Transfer Rate (%)** | **log K*_ow_*^a^** |
| --- | --- | --- | --- | --- | --- |
| 1-Methylnaphthalene | L | 2 | 2.10 | 67.79 | 3.87 |
| 2-Methylfluorene | L | 3 | 1.39 | 58.20 | 4.56 |
| 2-Methylnaphthalene | L | 2 | 2.97 | 74.82 | 4 |
| 2,3,5-Trimethylnaphthalene | L | 2 | 0.97 | 49.22 | 4.8 |
| 2,3,6-Trimethylnaphthalene | L | 2 | 0.95 | 48.62 | 4.73 |
| 2,6-Dimethylnaphthalene | L | 2 | 2.08 | 67.49 | 4.31 |
| Acenaphthene | L | 3 | 2.76 | 73.43 | 3.92 |
| Acenaphthylene | L | 3 | 1.02 | 50.39 | 3.93 |
| Anthracene | L | 3 | 2.32 | 69.90 | 4.45 |
| Benz[a]anthracene | H | 4 | 0.95 | 48.68 | 5.46 |
| Biphenyl | L | 2 | 2.85 | 73.99 | 4.01 |
| C1-Benzo[a]anthracenes/Chrysenes | NA | NA | 1.00 | 50.00 | N/A |
| C1-Biphenyls | L | 2 | 3.32 | 76.83 | N/A |
| C1-Fluoranthenes/Pyrenes | NA | NA | 0.25 | 19.81 | N/A |
| C1-Fluorenes | L | 3 | 1.78 | 64.09 | N/A |
| C1-Naphthalenes | L | 2 | 2.67 | 72.77 | N/A |
| C2 Phenanthrenes/Anthracenes | NA | NA | 0.29 | 22.65 | N/A |
| C2-Biphenyls | L | 2 | 10.84 | 91.56 | N/A |
| C2-Dibenzothiophenes | L | 3 | 5.38 | 84.32 | N/A |
| C2-Fluoranthenes/Pyrenes | NA | NA | 0.68 | 40.51 | N/A |
| C2-Fluorenes | L | 3 | 3.11 | 75.65 | N/A |
| C2-Naphthalenes | L | 2 | 1.81 | 64.46 | N/A |
| C3-Dibenzothiophenes | L | 3 | 1.06 | 51.42 | N/A |
| C3-Fluoranthenes/Pyrenes | NA | NA | 1.00 | 50.00 | N/A |
| C3-Fluorenes | L | 3 | 56.15 | 98.25 | N/A |
| C3-Naphthalenes | L | 2 | 1.14 | 53.26 | N/A |
| C3-Phenanthrenes/Anthracenes | NA | NA | 1.25 | 55.56 | N/A |
| C4-Dibenzothiophenes | L | 2 | 2.16 | 68.40 | N/A |
| C4-Fluoranthenes/Pyrenes | NA | NA | 1.29 | 56.39 | N/A |
| C4-Naphthalenes | L | 2 | 2.55 | 71.79 | N/A |
| C4-Phenanthrenes/Anthracenes | NA | NA | 2.93 | 74.59 | N/A |
| Chrysene | H | 4 | 4.89 | 83.02 | 5.81 |
| Dibenzothiophene | L | 2 | 17.02 | 94.45 | 4.38 |
| Fluoranthene | H | 4 | 0.74 | 42.50 | 5.16 |
| Fluorene | L | 3 | 1.44 | 59.09 | 4.18 |
| Naphthalene | L | 2 | 13.82 | 93.25 | 3.3 |
| Phenanthrene | L | 3 | 1.09 | 52.17 | 4.46 |
| Pyrene | H | 4 | 0.47 | 31.75 | 4.88 |

**^a^**According to ^1–4^.

**Table S3.** Diagnostic ratios for each killer whale (*O. orca*) sample.

| **Killer Whale ID** | **Ant/(Ant+Phe)** | **Flu/(Flu+Pyr)** | **BaA/(BaA + Chr)** |
| --- | --- | --- | --- |
| 10/01835, DFO 5646 Liver | 0.88 | 0.57 | 0.84 |
| 16-1664 Liver | 0.74 | 0.32 | 0.39 |
| 16-1664 SM | 0.4 | 0.38 | 0.17 |
| AHC 13-1550 Liver | 0.46 | 0.98 | 0.9 |
| AHC 14-5855 (J32) SM | 0.48 | 0.35 | 0.9 |
| AHC 14-5856 (J32 fetus) Liver | 0.38 | 0.48 | 0.39 |
| AHC 14-5856 (J32 fetus) SM | 0.67 | 0.46 | 0.63 |
| AHC 15-6931 Liver | 0.58 | 0.57 | 0.81 |
| AHC 16-1760 (L95) Liver | 0.29 | 0.37 | 0.39 |
| AHC 16-4828 Liver | 0.61 | 0.78 | 0.74 |
| AHC 16-6517 (J34) Liver | 0.16 | 0.39 | 0.39 |
| AHC 18-6458 Liver | 0.3 | 0.57 | 0.96 |
| L98 (Luna: case 06/00938) SM | 0.1 | 0.55 | 0.33 |
| T171 AHC 13-4290 Liver | 0.85 | 0.98 | 0.65 |

**Table S4.** Biometrics and descriptions of analyzed tissue in the 12 stranded killer whales (*O. orca*) collected in British Columbia, Canada, 2006-2018. SRKW = Southern Resident killer whale, NA = not available. Reprinted (adapted) with permission from {Lee, K. *et al.* Emerging contaminants and new POPs (PFAS and HBCDD) in endangered Southern Resident and Bigg’s (Transient) killer whales (*Orcinus orca*): *In utero* maternal transfer and pollution management implications. *Environ. Sci. Technol.* (2023) doi:10.1021/acs.est.2c04126}. Copyright {2023} American Chemical Society.

| **Killer Whale Identification (ID)** | **Recovery Date** | **Location** | **Age Category** | **Age Estimate (year)** | **Sex** | **Ecotype** | **Sample(s) Analyzed**  **(SM = skeletal muscle)** | **Carcass Condition Code** | **Body Condition Index (BCI)** | **Percent (%) Lipid** |
| --- | --- | --- | --- | --- | --- | --- | --- | --- | --- | --- |
| L98 (Luna: case 06/00938) | 2006-03-10 | Nootka Sound, Gold River, BC | Juvenile | 7 | Male | SRKW | SM | 2 | NA | 7.76 |
| 10/01835, DFO 5646 | 2010-05-04 | Sooke, BC | Neonate | 0.1* | Male | Bigg’s | Liver | 3 | NA | 3.65 |
| AHC 13-1550 | 2013-04-13 | Carmanah Beach, BC | Adult | NA | Female | Bigg’s | Liver | 4 | NA | 13.4 |
| T171 AHC 13-4290 | 2013-10-18 | Prince Rupert, BC | Adult | 29 | Female | Bigg’s | Liver | Late 3 | 0.56 | 10.4 |
| AHC 14-5855 (J32) | 2014-12-06 | Comox, BC | Adult | 18 | Female | SRKW | SM | 3 | 0.65 | 2.76 |
| AHC 14-5856 (J32 fetus) | 2014-12-06 | Comox, BC | Fetus | NA | Female | SRKW | SM + Liver | 3 | 0.73 | 5.9 (SM) + 4.48 (Liver) |
| AHC 15-6931 | 2015-12-25 | Tofino, BC | Neonate | 0.1* | Female | Bigg’s | Liver | 3 | 0.05 | 3.83 |
| 16-1664 | 2016-03-25 | Sooke | Neonate | 0.1* | Female | SRKW | SM + Liver | 3 | NA | 1.3 (SM) + 16.3 (Liver) |
| AHC 16-1760 (L95) | 2016-03-31 | Esperanza Inlet, BC | Adult | 20 | Male | SRKW | Liver | 4 | NA | 11.3 |
| AHC 16-4828 | 2016-09-15 | Pachena Bay, BC | Adult | 34 | Male | Bigg’s | Liver | 3 | 0.6 | 9.36 |
| AHC 16-6517 (J34) | 2016-12-20 | Sechelt, BC | Adult | 18 | Male | SRKW | Liver | 3 | NA | 4.32 |
| AHC 18-6458 | 2018-11-14 | Nootka Island, BC | Neonate | 0.1* | Unknown | Bigg’s | Liver | 3 | NA | 3.96 |

*All neonates were considered to be one month old (~0.1 year).

**Table S5.** List of significance (*p-*values, where *α* = 0.05) found between given variables. Pearson’s Product-Moment or Spearman Rank correlation was used to determine correlations while Welch’s Two Sample *t*-test or Wilcoxon Rank Sum Exact test was used for comparisons between confounding variables and PAH contamination. Note: SRKW = Southern Resident killer whale.

| **Correlation between percent (%) lipid (Table S1) and PAH concentration** | | | |
| --- | --- | --- | --- |
| **Contaminant** | | ***P*-value** | |
| 2,3,5-trimethylnaphthalene | | 0.03 | |
| C2-biphenyls | | 0.037 | |
| Fluorene | | 0.031 | |
| **Correlation between body condition indices (BCI) of all samples (note: BCI values were available for only six individual killer whales; n=6) and PAH concentration** | | | |
| **Contaminant** | | ***P*-value** | |
| Benz[a]anthracene | | 0.05 | |
| Biphenyl | | 0.041 | |
| C1-benzo[a]anthracenes/chrysenes | | 0.0086 | |
| C1-fluorantheses/pyrenes | | 0.043 | |
| C2 phenanthrenes/anthracenes | | 0.03 | |
| C2-fluoranthenes/pyrenes | | 0.014 | |
| C3 phenanthrenes/anthracenes | | 0.0065 | |
| C4-fluoranthenes/pyrenes | | 0.023 | |
| C4 phenanthrenes/anthracenes | | 0.047 | |
| **PAH Concentration differences between sex (male: n = 5, female: n = 6)**  (all significantly higher in males) | | | |
| **Contaminant** | ***P*-value** | | **Higher in** |
| C4-fluoranthenes/pyrenes | 0.037 | | Males |
| C4-naphthalenes | 0.047 | | Males |
| C4-phenanthrenes/anthracenes | 0.05 | | Males |
| Chrysene | 0.019 | | Males |
| Dibenzothiophene | 0.0016 | | Males |
| Fluoranthene | 0.045 | | Males |
| Fluorene | 0.036 | | Males |
| Naphthalene | 0.0016 | | Males |
| **PAH concentration differences between SRKW (n=3) and Bigg’s killer whale (n=4) calves (Fig S2)** | | | |
| **Contaminant** | ***P*-value** | | **Higher in** |
| 2-methylfluorene | 0.03 | | SRKW |
| Acenaphthylene | 0.045 | | Bigg’s |
| Benz[a]anthracene | 0.02 | | Bigg’s |
| Biphenyl | 0.037 | | SRKW |
| C1-fluoranthenes/pyrenes | 0.0021 | | Bigg’s |
| C2 phenanthrenes/anthracenes | 0.0025 | | Bigg’s |
| C2-dibenzothiophenes | 0.017 | | Bigg’s |
| C2-fluoranthenes/pyrenes | 0.019 | | Bigg’s |
| C3-dibenzothiophenes | 0.019 | | Bigg’s |
| C3-fluoranthenes/pyrenes | 0.033 | | Bigg’s |
| C3-fluorenes | 0.0078 | | Bigg’s |
| C3-phenanthrenes/anthracenes | 0.005 | | Bigg’s |
| C4-dibenzothiophenes | 0.024 | | Bigg’s |
| C4-fluoranthenes/pyrenes | 0.012 | | Bigg’s |
| C4-phenanthrenes/anthracenes | 0.011 | | Bigg’s |

**Table S6.** Raw wet weight contaminant concentration (ng/g ww) for each killer whale (*O. orca*) sample (SAMPLE ID). Data is not blank corrected. SM = Skeletal Muscle, ND = Not Detected at reporting limit.

See excel sheet Table S6.

# **References**

1. Achten, C. & Andersson, J. T. Overview of polycyclic aromatic compounds (PAC). *Polycycl. Aromat. Compd.* **35**, 177–186 (2015).

2. ChemIDplus. https://chem.nlm.nih.gov/chemidplus/ (2021).

3. ChemSpider. https://www.chemspider.com/ (2021).

4. PubChem. *Deparmtent of Health and Human Services, Government of the United States* https://pubchem.ncbi.nlm.nih.gov/ (2021).
